# Membrane Transporters for Bilirubin and Its Conjugates: A Systematic Review

**DOI:** 10.3389/fphar.2017.00887

**Published:** 2017-12-05

**Authors:** Jovana Čvorović, Sabina Passamonti

**Affiliations:** Department of Life Sciences, University of Trieste, Trieste, Italy

**Keywords:** bilirubin, membrane, transporter, assay, systematic review

## Abstract

**Background:** Bilirubin is a highly-hydrophobic tetrapyrrole which binds to plasma albumin. It is conjugated in the liver to glucuronic acid, and the water-soluble glucuronides are excreted in urine and bile. The membrane transporters of bilirubin diglucuronide are well-known. Still undefined are however the transporters performing the uptake of bilirubin from the blood into the liver, a process known to be fast and not rate-limited. The biological importance of this process may be appraised by considering that in normal adults 200–300 mg of bilirubin are produced daily, as a result of the physiologic turnover of hemoglobin and cellular cytochromes. Nevertheless, research in this field has yielded controversial and contradicting results. We have undertaken a systematic review of the literature, believing in its utility to improve the existing knowledge and promote further advancements.

**Methods:** We have sourced the PubMed database until 30 June 2017 by applying 5 sequential searches. Screening and eligibility criteria were applied to retain research articles reporting results obtained by using bilirubin molecules in membrane transport assays *in vitro* or by assessing serum bilirubin levels in *in vivo* experiments.

**Results:** We have identified 311 articles, retaining 44, reporting data on experimental models having 6 incremental increases of complexity (isolated proteins, membrane vesicles, cells, organ fragments, *in vivo* rodents, and human studies), demonstrating the function of 19 membrane transporters, encoded by either *SLCO* or *ABC* genes. Three other bilirubin transporters have no gene, though one, i.e., bilitranslocase, is annotated in the Transporter Classification Database.

**Conclusions:** This is the first review that has systematically examined the membrane transporters for bilirubin and its conjugates. Paradoxically, the remarkable advancements in the field of membrane transport of bilirubin have pointed to the elusive mechanism(s) enabling bilirubin to diffuse into the liver as if no cellular boundary existed.

## Introduction

Bilirubin (BR) is a tetrapyrrolic pigment found in plasma as an albumin-bound reversible complex (Jacobsen and Brodersen, 1983). It derives from catabolism of heme, released by both hemoglobin (75%) and cytochromes (25%), with a daily production of about 200–300 mg in a normal adult (Levitt and Levitt, 2014). The liver is the organ where BR is taken up, metabolized by glucurono-conjugation, and excreted into the bile (Sticova and Jirsa, 2013).

In the liver, the membrane transport mechanisms of BR have distinctive features, depending on the BR molecule (conjugated or not), the plasma membrane interface (vascular or biliary), and the bioenergetics of transport (to electrochemical equilibrium or not).

Considering sinusoidal uptake of BR, the liver has the capacity to take up as much as 50% of a BR load in a single pass (Stollman et al., 1983) by a process that shows substrate saturation and competitive inhibition by other organic anions, like indocyanine green and bromosulfophthalein (BSP) (Hunton et al., 1961; Scharschmidt et al., 1975; Bloomer and Zaccaria, 1976). This is evidence for a transporter-mediated process. Considering transport of conjugated BR from the liver parenchyma to the bile, it is rate-limiting, not inhibited by BR (Goresky, 1965), and energy-dependent (Nakatani et al., 1995).

Genetic diseases have shed light on the membrane transporters for BR and its conjugates (Erlinger et al., 2014; Keppler, 2014) The Dubin-Johnson syndrome is caused by mutations in the protein MRP2, encoded by *ABCC2*, which drastically reduce the capacity of the liver to excrete the conjugated pigment into the bile. The sister protein MRP3, encoded by *ABCC3*, is expressed at the sinusoidal membrane and pumps bilirubin diglucuronide (BDG) back into the blood, which leads to conjugated hyperbilirubinaemia. Rotor syndrome is characterized by defective reuptake of BDG from the blood into the liver by OATP1B1/OATP1B3, encoded by *SLCO1B1/1B3* (Keppler, 2014) and is known as a predominantly conjugated hyperbilirubinemia.

The Gilbert's syndrome is characterized by familial unconjugated hyperbilirubinemia, due to *UGT1A1* mutations causing defective conjugation of BR with glucuronic acid in the liver. However, defects of hepatic uptake transporters might contribute to pathogenesis, as pointed out in a study on subjects displaying the Gilbert's syndrome but no *UGT1A1* mutations (Skierka et al., 2013).

Technical, biological and conceptual issues have contrasted the progress of knowledge about the transporter-mediated diffusion of unconjugated bilirubin (UCB) across biological membranes. These are the insolubility and instability of BR in aqueous media (McDonagh, 2008), the very fast rate of transport (Billing and Black, 1969), the property of organic anion transporters, such as OATPs, to handle a large number of different drugs, metabolites or phytochemicals (Hagenbuch and Stieger, 2013) and, not least, a steady confidence in the occurrence of a single transporter protein performing this task.

Yet, transfer of BR across biological membranes remains one of the fundamental questions of cellular biology, at the basis of our understanding of the clinical biomarker bilirubinemia (Wang et al., 2006; Levitt and Levitt, 2014). The central question is about what makes it possible for the liver to take up 0.13–0.2 mg BR min^−1^.

Normal fluctuations of bilirubinemia have gained momentum because mild increases are related to reduction of cardiovascular disease risk, type-2 diabetes mellitus and other conditions (Kapitulnik and Maines, 2012). Thus, it would be important to understand if membrane transporters may participate in these beneficial fluctuations, if they transport other endogenous metabolites, or plant secondary metabolites that are in our diet, or drugs, or if they can be targeted by time-dependent protein reagents resulting in up- or down-regulation, and so many other questions that other systems have already successfully addressed.

We recognized that a comprehensive review on BR-specific membrane transporters is missing in the literature. In the attempt to fill this deficit, we have undertaken a systematic review of the literature, believing in its utility to both improve the existing knowledge and promote a rational planning of new investigations.

We addressed some fundamental research questions, common to any experimental study about membrane transport, such as the following.

### What transporters catalyze translocation of bilirubin across biological membranes?

This was a primary research question and one of the criteria for eligibility.

### What experimental model was used to assess bilirubin transport, either *in vivo* or *in vitro*?

It is important to have a survey of the methods used and the related results, possibly independently obtained by different research groups. It is a question relevant for the case of the reproducibility, usability and predictability of available research results. We searched for *protein, vesicles/liposomes, isolated cells, tissue fragments, isolated organs, in vivo animals, and clinical trials*.

### What bilirubin molecule was used in *in vitro* assays?

This was a primary research question and one of the criteria for eligibility. Both BR and its conjugated derivatives were considered, because both are transported by transporter-mediated mechanisms.

### What bilirubin concentrations were used in transporter activity assays?

The concentration conditions selected for the *in vitro* studies were clearly relevant for the quality of the data, not only because an assay that used physiological concentrations of transport substrate or modulator had a higher predictability power, but also because of chemical stability of BR.

### Was bilirubin used as a substrate or as a modulator of transporter activity?

Direct analysis of a transport substrate is the preferred approach in any assay. However, the poor water solubility of BR (i.e., the albumin-free, unconjugated species), its relevant physiological concentration in the nM range, and its time-dependent chemical decay limit the versatility of BR-based transport assays. By exploiting the principles of inhibition kinetics, it is possible to use an alternate transport substrate in the presence of BR as a transporter modulator (typically a competitive inhibitor) and so to estimate the properties of the transporter-BR interaction(s).

### What competing molecules interfere with bilirubin transporters?

Knowledge of the molecules competing with BR for membrane transport sheds light on both fundamental aspects of BR metabolism and its value as a serum biomarker. Furthermore, this question concerns methodological issues, since some of these competing molecules might be used in activity assays of BR-specific membrane transporters.

## Materials and methods

We have implemented the PRISMA guidelines to retrieve, select and analyze primary research articles reporting results on BR membrane transporters (Shamseer et al., 2015).

### Identification of articles in public database

#### Information sources

We have chosen to search only PubMed, because it is the benchmark repository of biomedical literature. Though it can be considered that different databases have different algorithms to process queries and apply filters, this review has disregarded the point of managing, comparing and discussing differences in the performance of search process among different databases.

We searched PubMed in four sequential search steps, as described in Table 1.

**Table 1:** Search strategy.

**#**	**Search**	**Rationale**
1st	Bilirubin AND membrane AND transporter AND assay	Bilirubin is the tested molecule used in biological assays that measure the activity of a given membrane transporter.
2nd	Bilirubin AND membrane AND carrier protein AND assay	Search #2 is the same as search #1, except for using the once popular MESH term Carrier protein instead of Transporter.
3rd	Bilirubin AND membrane AND organic anion transporter AND assay	Search #3 is the same as search #1, except for using the MESH term Organic Anion Transporter instead of Transporter. Organic anions include bilirubin.
4th	OATP OR MRP OR MDR OR bilitranslocase AND bilirubin AND assay	Search #4 is a query of articles reporting bilirubin-based biological assays of transporters identified by search#1 (i.e., *OATP OR MRP OR MDR OR bilitranslocase*).
5th	Manual	Retrieval of articles that have escaped query-based searches, in spite of being indexed by MeSH terms such as “transporter” and “bilirubin”.

#### Search strategy

The time window was open, with the first paper dated 1970–June 2017. Filters were not applied. The term transporter was chosen because it is a standard term that defines a protein entity associated with membrane transport function, corresponding to the MeSH Heading Membrane Transport Proteins (U.S. National Library of Medicine).

The output of each search step was a set of automatically identified articles. The second, third and fourth searches contained duplicate articles that were immediately removed. The fifth search was manual.

### Selection of eligible articles

#### Selection strategy

The articles identified by the sequential search steps were selected by reading the abstract and/or the full text. Criteria for retaining (IN) or discarding (OUT) are detailed in Table 2. As a result of screening, selected articles underwent a more stringent eligibility check, which retained articles eligible for the analysis and classification of their study features.

**Table 2:** Selection strategy.

**Phase**	**Text**	**In**	**Out**	**Results**
Screening	Abstract (with Materials and Methods, if needed)	Primary/original research articles Using mammalian experimental models for studying membrane transporters Using bilirubin	Not in English Reviews Not performed in mammalian models Not using mammalian genetic material Not reporting results on bilirubin analysis in biological or physiological fluids Not reporting results on biological transport assays	Articles screened for further eligibility check
Eligibility check	Full text	The article section “Materials and Methods” describes: (a) a biological assay to assess membrane transport activity using bilirubin as either substrate or modulator; (b) chemical analysis of bilirubin in bio-fluids or tissue extracts. The article section “Results” (including Figures and Tables) reports experimental data about transport and distribution of bilirubin in compartments separated by biological membranes.	Bilirubin transport was just hypothesized on the basis of existing literature Bilirubin was tested as inducer of the expression of membrane transporters, without experimental proof of change in bilirubin transport activity or distribution in compartments Mutated forms of membrane transporters were tested, without experimental proof of change in bilirubin transport activity	Included articles as eligible for the analysis of their study features (STEP 3 of the systematic review)

## Results

### Literature search and selection

**Figure 1:**
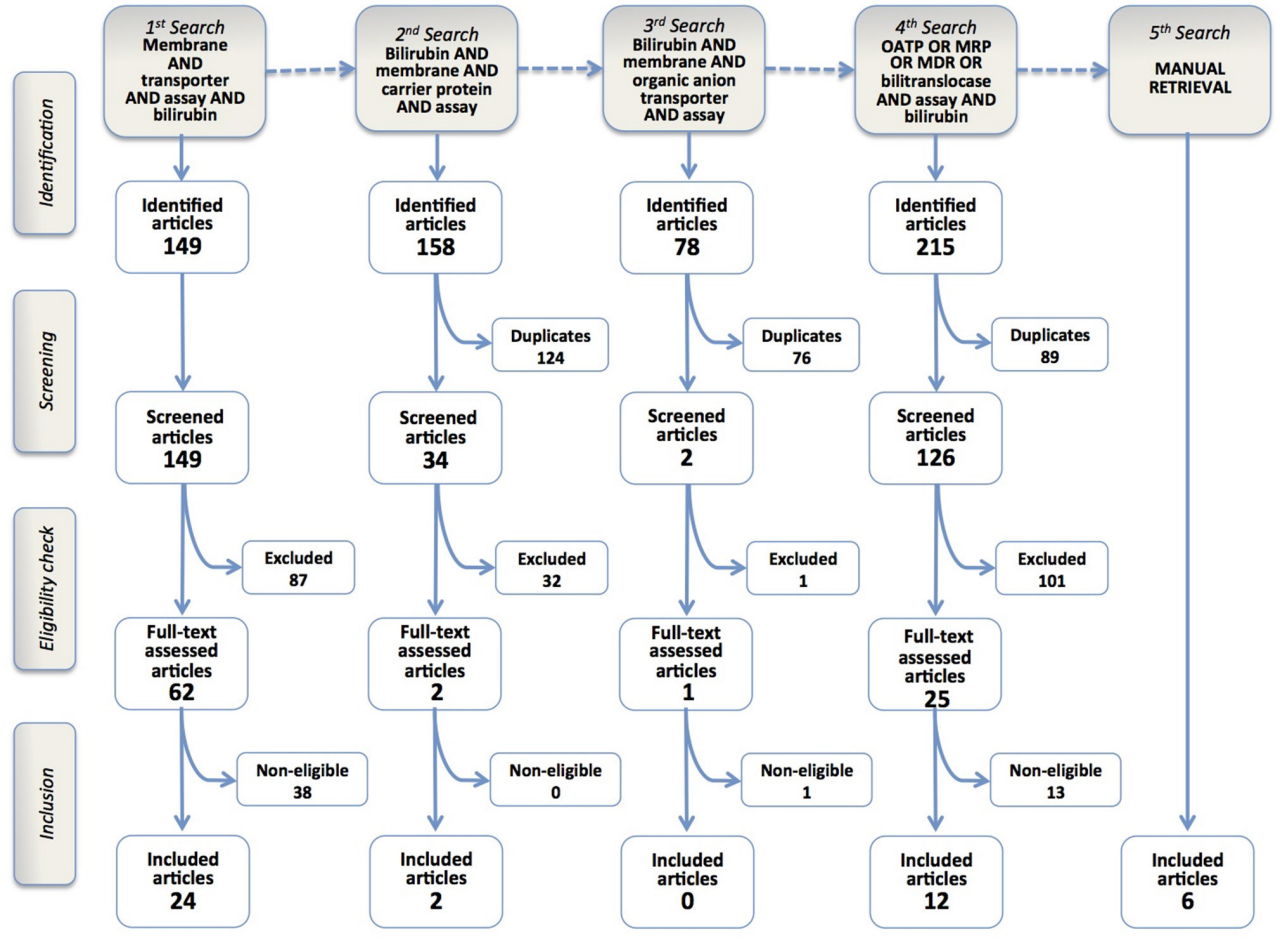
Flowchart of the results of the literature search strategy and implementation of inclusion criteria. Four query-based searches and a manual one resulted in the inclusion of 44 articles.

Figure 1 shows the literature search strategy and the results obtained at each of the five steps of the selection process.

The 1st search identified 149 article titles. Screening and eligibility check enabled to include 24 articles in the review.

After this first step, we realized the limitations of the search query, since some of the seminal papers on BR membrane transport were not present among identified records (e.g., Stremmel et al., 1983; Cui et al., 2001; Briz et al., 2003). Therefore, we decided to perform a sequence of query-based searches, in order to refine our search strategy and possibly retrieve missing articles.

In the 2nd and 3rd search phases (Figure 1), the term “transporter” was substituted with either “carrier protein,” a popular term in pre-genomic times, or the more restrictive term “organic anion transporter.” We retrieved 158 and 78 records, respectively, but only 2 were included in the final analysis. Nevertheless, some classic papers were still missing (Cui et al., 2001; Briz et al., 2003), raising the concern that some others might have been left behind.

The 4th search phase was more straightforward, using the names of BR membrane transporter proteins described in previously retrieved articles, such as OATP, MRP, MDR or bilitranslocase. It identified 215 articles, of which 14 were included in the review.

The last phase was the manual retrieval of some articles that escaped the prior searches. There is no obvious reason why two articles (Cui et al., 2001; Briz et al., 2003), which are considered milestones in transporter-mediated BR uptake and determined a consolidated consensus on the role of OATP1B1 and OATP1B3 in hepatic BR uptake (Stieger and Hagenbuch, 2014), could not be retrieved, in spite of the fact that their MESH terms listed both “transporter” and “bilirubin.” Similarly, other papers using BR and its conjugates as a transport substrate in accurate biochemical studies (Jedlitschky et al., 1997; Kamisako et al., 1999; Pascolo et al., 2001; Rigato et al., 2004) remained out. Other articles, reporting *in vivo* studies with drug transporter-deficient mouse models with hypebilirubinemic phenotypes (Chen et al., 2008; Lu et al., 2008) were lost but, in these cases, their MeSH terms did not mention BR, which was a constitutive element of all search queries of this review. The approach to manually retrieve these articles was to check the list of references of the included articles, or of reviews on drug and BR transporters. The manual retrieval of articles was however kept to the least possible extent, even if this implied the risk of overlooking some articles. In fact, manual retrieval is neither systematic nor automatic, i.e., it is not reproducible, since it stems from individual experience, curiosity or perspicacity. By contrast, this review aims at offering a universal “baseline” of current knowledge on BR membrane transporters and a method to update it and so measure the future progress in the field.

### Features of bilirubin membrane transport studies

The 44 papers to be analyzed were then classified in two groups—*in vitro* and *in vivo* studies, according to the experimental model used to study BR transport. These studies are briefly described below, with limited reference to other items of literature.

### *In vitro* studies on membrane transport of bilirubin

The list of *in vitro* transport studies is reported in Table 3. Assays used BR molecules either as transport substrates or as transport modulators added to the assay mixture containing dyes, drugs or metabolites. The dye sulfobromphthalein (BSP), used in hepatic functional tests (Burra and Masier, 2004), has been applied in pioneering pharmacokinetic experiments that demonstrated the capacity of BR to inhibit BSP clearance in rats (Scharschmidt et al., 1975).

**Table 3:** Features of included articles reporting *in vitro* studies.

**References**	**Experimental model description**	**Bilirubin molecule**	**[Bilirubin]**	**Bilirubin-transporter interaction**	**Competing substance**	**Transporter**	**Search #**
Wolkoff and Chung, 1980	Protein isolated from rat liver plasma membrane	Bilirubin	1.5–20 μM	Substrate	BSP	OABP	1st
Stremmel et al., 1983	Rat liver plasma membrane vesicles	Bilirubin	0.2–8 μM	Substrate	BSP	Not defined—55 kDa membrane protein	2nd
Adachi et al., 1990	Rat liver plasma membrane vesicles	bis-glucuronosyl bilirubin, bilirubin	50 μM	Substrate; modulator	Bilirubin; BSP	Unknown	1st
Passamonti and Sottocasa, 1990	Rat liver plasma membrane vesicles	Bilirubin	8–40 nM	Modulator	BSP	bilitranslocase	1st
Stremmel and Diede, 1990	HepG2 cells (human hepato-carcinoma)	bis-glucuronosyl bilirubin	4–48 μM	Substrate	BSP	Not defined—55 kDa membrane protein	2nd
Passamonti et al., 1997	Rat liver plasma membrane vesicles	Bilirubin	2–20 nM	Modulator	BSP	bilitranslocase	1st
Jedlitschky et al., 1997	Plasma membrane vesicles from HeLa, HeLa-MRP1 and HepG2 cells; canalicular membrane vesicles from livers of normal Wistar rats and from Mrp2-deficient GY/TR– mutant rats	Mono-and bis-glucuronosyl bilirubin	0.5 μM	Substrate	Leukotriene C4	MRP1, MRP2, mrp2	5th
Kamisako et al., 1999	Membrane vesicles from HEK293 stably expressing rat or human MRP2	[^3^H] Mono-and bis-glucuronosyl bilirubin	0.5 μM	Substrate	Leukotriene C4, 17b-glucuronosyl estradiol, cyclosporin A	MRP2, mrp2	5th
(König et al., 2000)	HEK293 stably expressing OATP1B1	Mono-glucuronosyl bilirubin	80 nM	Substrate		OATP1B1	1st
(Cui et al., 2001)	HEK293 permanently expressing OATP1B1 or OATP1B3	[^3^H] Bilirubin, [^3^H] Mono- and [^3^H] Bis-glucuronosyl Bilirubin	1 μM	Substrate	BSP	OATP1B1, OATP1B3	5th
Pascolo et al., 2001	BeWo cells (human placental trophoblast)	[^3^H] Bilirubin	5–100 nM	Substrate	BSP, indomethacin, taurocholate, MK571	MRP1, MRP5	5th
Wang et al., 2003	HeLa and HEK293 cells expressing OATP1B1	Bilirubin	1 μM	Substrate		OATP1B1	4th
Briz et al., 2003	Xenopus laevis oocytes expressing OATP1B1 or OATP1B3	[^3^H] Bilirubin	0–260 nM	Substrate		OATP1B1, OATP1B3	5th
		Bilirubin	100 μM	Modulator	17β-D-glucuronosyl oestradiol	OATP1B1, OATP1B3	
Lee et al., 2004	Membrane vesicles from MDCKII stably expressing MRP3	Mono-and bis-glucuronosyl bilirubin	12 nM (MGB), 9.5 nM (BGB)	Not characterized		MRP3	1st
Rigato et al., 2004	Membrane vesicles from MDCKII and MDCKII stably expressing MRP1 and MRP2	[^3^H] Bilirubin	6–72 nM	Substrate		MRP1, MRP2	5th
		Bilirubin	100 nM	Modulator	Leukotriene C4	MRP1, MRP2	
Passamonti et al., 2005b	HepG2 cells	Bilirubin	50 nM	Substrate	Nicotinic acid, BSP	bilitranslocase	1st
Calligaris et al., 2006	Primary mouse embryo fibroblasts from wild type and Mrp1 (–/–) mice	Bilirubin	4–140 nM	Substrate		Mrp1	4th
Maestro et al., 2010	Ea.hy926 cells (human vascular endothelium)	Bilirubin	50 nM	Modulator	Quercetin	bilitranslocase	1st
Nakanishi et al., 2012	Sandwich-cultured rat primary hepatocytes	Bilirubin	20 μM, 50 μM	Modulator	CDF (5-(and 6)-carboxy-2′,7′-dichlorofluorescein)	MRP2	1st
Terdoslavich et al., 2012	Human and rat liver precision-cut slices	Bilirubin	50 nM	Modulator	BSP	Bilitranslocase	1st
Chu et al., 2015	HEK293 cells expressing cynomolgus OATP1B1 and OATP1B3	[^3^H] bilirubin	40 nM	Substrate	Rifampin	OATP1B1, OATP1B3	1st
Matsushima et al., 2017	Inside-out rat erythrocyte membrane vesicles	bilirubin	10 μM	Substrate	2,4-dinitrophenyl-S-glutathione	MRPs	1st
	Washed rat erythrocytes						

#### Studies with bilirubin as substrate in uptake studies

The earliest attempts at identifying the membrane transporter mediating the uptake of BR into the liver include a study (Wolkoff and Chung, 1980) on a BSP-binding membrane protein, named Organic Anion Binding Protein (OABP). It was isolated from rat liver sinusoidal plasma membrane vesicles and the direct binding with BR was measured by fluorescence spectrometry, which led to the estimation of its dissociation constant *K*_d_ = 20 μM. Further cloning studies clarified that this protein was identical to a segment of the β-subunit of F1-ATPase (Goeser et al., 1990), which is well-known to have an extra-mitochondrial localization (Mangiullo et al., 2008). A dimeric BSP- and bilirubin-binding glycoprotein (BBBP) was also isolated from rat liver plasma membranes, considered to be probably related to OABP (Stremmel et al., 1983), but distinct from a third BR- and BSP-binding protein known as bilitranslocase (BTL) (Tiribelli et al., 1978). An antibody targeting BBBP inhibited the cellular uptake of BR, BDG, and BSP in the human hepatoma cell line HepG2, confirming the transporting function of this membrane protein (Stremmel and Diede, 1990), and its distinct nature from bilitranslocase (Torres et al., 1993).

The discovery of the cDNA encoding for Organic Anion Transporting Polypeptides (Jacquemin et al., 1994) opened the possibility to investigate the transport properties of members of this superfamily of solute transporters. A study (Cui et al., 2001) showed that [^3^H]BR could be transported into human embryonic kidney cells (HEK293) stably transfected with OATP2 (OATP1B1), but not with OATP8 (OATP1B3). The apparent K_m_ was calculated to be 160 nM. OATP1B1-mediated transport of BR was inhibited by BSP. These results were however questioned after assessing uptake of [^3^H]BR into HeLa cells expressing OATP2 (i.e., OATP1B1) (Wang et al., 2003), showing that it was not different from uptake in control cells. Yet, the same transfected cells performed a marked and specific increase in uptake of BSP. It was deduced that the data reported by Cui et al. should be ascribed to unspecific binding of [^3^H]BR on the cell membrane(s) (Wang et al., 2003). However, specific uptake of [^3^H]BR into *Xenopus* oocytes expressing OATP1B1 and OATP1B3 was also reported (Briz et al., 2003).

A more recent study (Chu et al., 2015) explored the possibility to measure uptake of [^3^H]BR (40 nM) into HEK293 cells stably transfected with cynomolgus OATP1B1 and OATP1B3. Though uptake of [^3^H]BR was measurable, uptake activity was too low to use this kind of assay to kinetically characterize BR transport or to screen transporter-drug interactions.

Another study was done on cultures of human adenocarcinoma cell line (HepG2). BR was added to the extracellular medium at 50 nM and its uptake into the cell monolayer was followed for 1.5 min by sampling and measuring the pigment via thermal lens spectrometry (Passamonti et al., 2005b), which exploits the thermal lens property of the pigment in solution to estimate its concentration (Franko, 2008). This analytical approach enabled to measure the progress of transfer of BR from the albumin-free medium to the cell monolayer, showing a time pattern similar to that recorded in perfused rat livers (Stollman et al., 1983). The study showed that a polyclonal antibody targeting an extracellular domain of a membrane protein bilitranslocase (Battiston et al., 1998) nearly abolished the cellular uptake of BR.

#### Studies with bilirubin as substrate in efflux studies

Besides the significance of BR uptake mechanisms, cellular efflux mechanisms that favor the extrusion of BR have a fundamental importance *in utero*. In facts, BR formed during fetal development is transferred to the maternal circulation across the cellular barrier provided by the placental trophoblast.

MRP1 is expressed at the level of the human placenta where it pumps a variety of xeno- and endobiotics back to the maternal blood (St-Pierre et al., 2000). It was identified as the most likely player in efflux of BR from cultured human trophoblast cells (BeWo cell line) used as a model of human placenta (Pascolo et al., 2001). Efflux occurred at the apical side, which is in contact with maternal blood. This study also assessed the kinetics of BR uptake into these cells, finding that it was not inhibited by taurocholate, a standard substrate of OATPs. Thus, the trophoblast cellular barrier ensures the transfer of BR from the fetal to the maternal circulation. The kinetic details of MRP1-dependent BR transport were obtained in a study using plasma membrane vesicles prepared from human cells transfected with MRP1 (K_m_ = 10 nM and V_max_ = 100 pmol min^−1^ mg^−1^ of protein) (Rigato et al., 2004). To characterize the role of Mrp1 in cellular efflux of BR and its protective effect against BR-induced cytotoxicity, mouse embryo fibroblasts from either wild type or Mrp1 knockout mice were used as a model (Calligaris et al., 2006). After 4 h of exposure, [^3^H]BR accumulated more in Mrp1^−/−^ than in wild type fibroblasts, under all conditions (40, 80 and 140 nM). Cellular ATP levels decreased in Mrp1^−/−^ cells but not in normal cells. BR (20–160 nM) was increasingly cytotoxic in both cell lines, with a stronger effect in mutant cells.

#### Studies with bilirubin as modulator

The effect of BR as modulator of transporter activity has been tested in several assays with model substrates, thus circumventing the technical difficulties of analysing BR at very low concentrations (<0.1 μM) and yet taking advantage of detailed kinetic analysis of transporter-BR interaction. Using sinusoidal rat liver plasma membrane vesicles, BR (50 or 100 μM, added at pH 7.8) was shown to inhibit BDG transport (Adachi et al., 1990) or BSP transport (Passamonti and Sottocasa, 1990).

A complex experiment, aimed at quantitatively assess the power of BR to protect from time-dependent inhibition of bilitranslocase caused by the serine-specific reagent PMSF, enabled to obtain an apparent dissociation constant (K_d_ = 2.1 nM) of the bilitranslocase-BR complex (Passamonti et al., 1997). The same kinetic method was used to characterize the interaction of bilitranslocase with anti-sequence antibodies, obtaining the same results (Battiston et al., 1998). The ability of BR to inhibit BSP uptake was also observed with liver slices obtained from either human or rat liver; such inhibition was found to overlap with that due to bilitranslocase antibodies, but not with taurocholate, which is a substrate of OATPs but not of bilitranslocase (Terdoslavich et al., 2012). BR was also shown to inhibit the uptake of quercetin (a flavonoid molecule) into cells of the vascular endothelium (cell line Ea.hy926), expressing bilitranslocase, as assessed using antibodies (Maestro et al., 2010).

BR was also found to inhibit uptake of 2,4-Dinitrophenyl-S-glutathione, a standard substrate of MRP1, MRP2 and MRP5, in washed erythrocytes and their inside-out vesicles (Matsushima et al., 2017).

#### Studies with bilirubin conjugates, used as substrates or modulators

These studies investigated the mechanism(s) responsible of either transport of conjugated BR into the bile canaliculus or their reflux from the liver into the blood.

In rat liver sinusoidal plasma membrane vesicles, uptake of bilirubin glucuronides has been characterized as sodium-independent, electrogenic and competitively inhibited by both BSP and BR (Adachi et al., 1990). Though this function has been left without further molecular characterization, it seems that it could be performed by OATPs expressed on the sinusoidal membrane. In fact, an experiment using [^3^H]BMG in HEK293 cells, transfected with the “SLC21A6 gene” and expressing “OATP2” protein (OATP1B1) (König et al., 2000), showed the involvement of this liver sinusoidal membrane transporter in the ATP-independent efflux to the blood of BMG and other organic anions. Later on, the same cell system was used to show that OATP1B1 transported [^3^H]BR conjugates (Cui et al., 2001).

ATP-dependent efflux of BR conjugates also occurs at the sinusoidal membrane surface of hepatocytes, as shown in a study (Lee et al., 2004) that used plasma membrane vesicles obtained from the cell line MDCKII stably transfected with MRP3. The data support the concept that MRP3-mediated efflux of bilirubin glucuronides and other cholephilic compounds is a mechanism underlying the reflux of bile components from the liver to the blood, in situations of cholestasis.

Considering the ATP-dependent transporters expressed at the canalicular side of the liver cells, one of the pioneering studies used various plasma membrane preparations expressing human MRP1 and MRP2 and rat Mrp2, aiming at characterizing the ATP-dependent transport of both bilirubin mono-glucuronide (BMG) and BDG. Furthermore, both conjugates were demonstrated to inhibit leukotriene transport (Jedlitschky et al., 1997), which is mediated by the same ATP-dependent primary active transporters, with IC_50_ values 0.1–0.7 μM, depending on the type of membrane preparation. A study (Kamisako et al., 1999) using membrane vesicles prepared from human cells transfected with either rat Mrp2 or human MRP2 provided the kinetic details of ATP-dependent transport of BMG and BDG (K_m_ = 0.7–0.9 μM and V_max_ = 255–321 pmol min^−1^ mg^−1^ of protein). A study using sandwich-cultured rat hepatocytes forming bile canaliculi (Nakanishi et al., 2012) confirmed that bilirubin glucuronides could inhibit the Mrp2-mediated secretion of a fluorescein compound in the canaliculi, as detected by quantitative time-lapse imaging.

### *In vivo* studies on membrane transport of bilirubin

Animal models with defective expression of membrane transporters have essentially contributed to the elucidation of the mechanisms of BR uptake into the liver, excretion into the bile, and reflux from the liver to the blood. Table 4 lists *in vivo* transport studies.

**Table 4:** Features of included articles reporting *in vivo* studies.

**References**	**Species**	**Experimental model**	**Treatment**	**Tissue/Biofluid**	**Transporter**	**Search #**
Watchko et al., 1998	Mouse	Mdr1^−/−^	Single i.v. dose of bilirubin (50 mg/kg)	Whole-brain, serum	P-gp	1st
Hankø et al., 2003	Rat	Young adult rats	Single i.v. dose of bilirubin (50 mg/kg)	Whole-brain, serum	P-gp	1st
Belinsky et al., 2005	Mouse	Mrp3^−/−^	Bile duct ligation	Serum	Mrp3	1st
Hirouchi et al., 2005	Rat	Eisai hyperbilirubinuria rats	Transfection of MRP2	Serum, bile	MRP2	1st
Itani et al., 2005	Rat	Eisai hyperbilirubinuria rats	Transfection of MRP2	Serum	MRP2	4th
Chu et al., 2006	Mouse	Mrp2^−/−^		Serum, urine, bile	Mrp2	1st
Mennone et al., 2006	Mouse	Mrp4^−/−^	Bile duct ligation	Serum	Mrp4	1st
Narvaiza et al., 2006	Mouse	Wild type mice	Silencing of Abcc2 by shRNAs	Serum	Mrp2	4th
Nishiya et al., 2006	Rat	Eisai hyperbilirubinuria rats	Single oral dose of tienilic acid (300 mg/kg)	Serum, bile	Mrp3	1st
Zelcer et al., 2006	Mouse	Mrp3^−/−^	Bile duct ligation	Serum	Mrp3	4th
Maher et al., 2008	Mouse	Wild type mice	Single i.p. dose of perfluorodecanoic acid (80 mg/kg)	Serum	Mrp3, Mrp4	4th
Zaher et al., 2008	Mouse	Oatp1b2^−/−^		Serum	Oatp1b2	4th
Mennone et al., 2010	Mouse	Bcrp^−/−^	Bile duct ligation	Serum	Bcrp	1st
van de Steeg et al., 2010	Mouse	Oatp1a/1b		Serum, bile, urine	Oatp1a1, Oatp1a4, Oatp1a5, Oatp1a6, Oatp1b2	4th
Vanwijngaerden et al., 2011	Human	Intensive care unit patients		Serum	NTCP, OATP1B1, OATP1B3, MRP3, MRP4, BSEP, MRP2, MDR1, MDR3	4th
Gong et al., 2011	Mouse	Oatp1a1^−/−^ and Oatp1a4^−/−^		Serum	Oatp1a1, Oatp1a4	4th
Hayashi et al., 2012	Human	Ornithine transcarbamylase deficiency patients	Treatment with 4-phenylbutyrate	Serum	MRP2	1st
Scheer et al., 2012	Mouse	Mrp2^−/−^	Transfection of MRP2	Serum, urine	MRP2	1st
van de Steeg et al., 2012	Mouse	(i) Oatp1a/1b^−/−^, Mrp3^−/−^, (ii) Oatp1a/1b^−/−^, Mrp2^−/−^, (iii) Oatp1a/1b^−/−^, Mrp3^−/−^, Mrp2^−/−^	Transfection of OATP1B1, OATP1B3 in Oatp1a/1b^−/−^	Serum	MRP2, MRP3, OATP1B1, OATP1B3	1st
van de Steeg et al., 2013	Mouse	Oatp1a/1b^−/−^	Transfection of OATP1B1, OATP1B3, OATP1A2	Serum, bile, urine	OATP1A2, OATP1B1, OATP1B3	4th
Chu et al., 2015	Monkey	Cynomolgus monkeys	Single oral dose of rifamycin (18 mg/kg)	Serum	OATP1B1, OATP1B3	1st
Watanabe et al., 2015	Rat	Young adult rats	Single i.v. injection of rifampicin (5, 20, or 80 mg/kg)	Serum	rOatps, rMrp2	4th
de Waart et al., 2016	Mouse	ATP11C^−/−^		Serum	Oatp1b2, Oatp1a1, Oatp1a4	1st

#### Sinusoidal uptake of bilirubin

Various murine models with disrupted expression of hepatic OATP transporters have been created, serving the primary need to assess their contribution to drug transport and interactions. The phenotype of these murine models has been assessed, by monitoring serum bilirubinemia, together with other biomarkers of liver pathology. Indeed, the knockout models displayed no sign of liver histopathology (Chen et al., 2008; Lu et al., 2008; Zaher et al., 2008; Gong et al., 2011) and no major change in expression of other influx or efflux transporters and P450 enzymes (Chen et al., 2008) or UDP-glucuronosyltransferase 1a1 (van de Steeg et al., 2010). In some cases, however, compensatory overexpression of other SLC transporters was noted (Lu et al., 2008).

The model mice Oatp1a1^−/−^ and Oatp1a4^−/−^ had normal clinical chemistry, i.e., no elevation of serum BR, showing that neither conjugated nor unconjugated bilirubin (UCB) is a substrate for these transporters. By contrast, sizeable changes in uptake and pharmacokinetics of some cholephilic compounds, such as estradiol 17-glucuronide (E2-17G), estradiol 3-glucuronide (E2-3G), and taurocholate, but not digoxin, were observed (Gong et al., 2011).

In rodent species, Oatp1b2 is the single ortholog that is most similar to both OATP1B1 and OATP1B3 expressed in human. The Oatp1b2^−/−^ mouse exhibited significant loss of function with respect to drug transport, such as phalloidin and microcystin-LR (Lu et al., 2008), rifampicin, rifamycin SV, lovastatin, simvastatin, pravastatin and cerivastatin (Chen et al., 2008; Zaher et al., 2008). All studies reported just mild and stable hyperbilirubinemia, mostly due to conjugated BR (Lu et al., 2008; Zaher et al., 2008). This suggests that, under normal conditions, Oatp1b2 mediates the reuptake of conjugated BR from the blood into the liver.

A mice mutant (Slco1a/1b^−/−^ mice), functionally deficient of all 5 Slco1a (Oatp1a1,−1a4, 1a5, and−1a6) and Slco1b2 (Oatp1b2) genes, was developed to examine the joint role of these Oatp members, expressed at the absorbing surface of liver, intestinal and kidney cells, in drug pharmacokinetics and drug-drug interactions (van de Steeg et al., 2010). The only effect that loss of these proteins caused was marked hyperbilirubinemia (>40-fold), with 95% of this increase due to conjugated BR. UCB increased by 2.5 times, together with unconjugated bile acids (13-fold). Though bile flow was unchanged, BR excretion was half than in wild types. Altogether, these data are consistent with the view that a substantial efflux of conjugated BR by sinusoidal Mrp3 occurs, and the loss of the influx pathway caused by the cluster of Oatp1a/1b is the main cause of the observed elevated levels of conjugated BR in serum. However, this model left unanswered the question regarding the mechanisms that ensure transfer of UCB from the blood into the liver (van de Steeg et al., 2010). When this knockout mouse model was transfected with the human genes encoding for OATP1A2, OATP1B1 or OATP1B3, total and conjugated BR in serum was partially (with OATP1A2) or completely normalized (with OATP1B1 and OATP1B3) (van de Steeg et al., 2013).

Another recent study combined both genetic studies in humans and functional studies in mouse models (van de Steeg et al., 2012) to describe the mechanistic basis of Rotor syndrome (benign hereditary conjugated hyperbilirubinemia). Using knockout mice deficient in one, two or all three genes encoding for Oatp1a/1b, Mrp 2 and Mrp3, the authors demonstrated the sequential involvement of Mrp3, which promotes excretion of BR conjugates in the blood at the level of upstream hepatocytes, and that of Oatp1a/1b, which mediates their reuptake in the downstream hepatocytes. Thus, the plasma bilirubin glucuronide levels were significantly increased in knockout mice deficient of Oatp1a/1b alone or both Oatp1a/1b and Mrp2. By contrast, in mice deficient of both Oatp1a/1b and Mrp3 these levels markedly decreased. To strengthen these findings and understand whether the corresponding human proteins act in the same way, Oatp1a/1b–deficient mice were transfected so to generate transgenic mice with liver-specific expression of human OATP1B1 or OATP1B3. In both models, conjugated BR levels were normalized suggesting the reabsorption of bilirubin glucuronides by OATP1B1 and OATP1B3. These data indicate the existence of a corresponding liver-blood shuttling loop in humans with MRP3, OATP1B1 and OATP1B3 as key players in maintaining BR blood levels stable.

The ATP11C-deficient mouse is a model that displays conjugated hyperbilirubinemia and hypercholanemia, as a consequence of the defect of this P-type ATPase that catalyzes the exchange of membrane phospholipids, also known as flippase. Flipping membrane phospholipids is an essential function in bile formation. ATP11C deficiency caused decreased expression of Oatp1b2, Oatp1a1, and Oatp1a4 (de Waart et al., 2016), thus simulating the role of Oatp1a/1b-knock-out mice (van de Steeg et al., 2010) and confirming that these Oatps contribute to hepatic reuptake of conjugated BR from the blood.

Consistently with the observation that transporter-deficient rodent models have no sign of liver pathology, except for hyperbilirubinemia, drugs that are transported by OATP and/or MRP transporters are expected to cause changes in bilirubinemia, in the absence of other signs of liver toxicity. A pharmacokinetic test done in rats with rifampicin, known for its potent and selective inhibition of Oatp transporters and a less potent effect on Mrp2, showed total serum bilirubin to increase by 62.8 ± 12.2% and 78.3 ± 16.0% at 1 h after administration of 20 and 80 mg/kg rifampicin, respectively, and these changes were due to conjugated BR (Watanabe et al., 2015). These results established the utility of serum BR as a sensitive and fast-responding biomarker of drug-transporter(s) interactions in pre-clinical studies. Nevertheless, a thorough analysis of a human hyperbilirubinemia dataset and the respective OATP1B1 and OATP1B3 inhibition predictions (Kotsampasakou et al., 2017a) indicated no strong association between OATP inhibition and hyperbilirubinemia, either for humans or for animals (Kotsampasakou et al., 2017b).

#### Excretion of conjugated bilirubin into the bile

In humans, active transport of BDG in the bile is mediated by MRP2, as proven in above-mentioned *in vitro* studies (Jedlitschky et al., 1997; Kamisako et al., 1999). Mutations of this gene are known to cause the autosomal recessive Dubin-Johnson syndrome with mild conjugated hyperbilirubinemia. The Eisai hyperbilirubinemic rat (Kawaguchi et al., 1994) has a hereditary loss of Mrp2 function and is therefore an experimental model of the Dubin-Johnson syndrome.

In Eisai hyperbilirubinemic rats, i.v. injection of recombinant adenoviruses containing MRP2 resulted in the simultaneous expression of MRP2 at the hepatic canalicular membrane, recovery of normal biliary excretion of DBSP (dibromosulfophtalein) and a marked decrease of serum direct (conjugated) BR (Hirouchi et al., 2005). Similar results were obtained using a different transfecting approach consisting in an MRP2 protein-expression plasmid vector, wrapped with the hemagglutinating virus of Japan envelope protein. One day after the i.v. injection of this kit, the expression of MRP2 in the liver canalicular membrane of Eisai hyperbilirubinemic rats was normalized. This entailed normalization of expression levels of Oatp1 and Oatp2 that are downregulated in this rat strain and of MRP3 that is upregulated. As a result, serum total and conjugated BR dropped within 24 h post-transfection (Itani et al., 2005).

Adenovirus was also used as a vector to transfect mice with short hairpin RNAs targeting Mrp2 (Abcc2). This treatment caused marked down-regulation of Abcc2 mRNA and a marked increase of total serum BR. These effects lasted several days, until the injected shRNAs were degraded (Narvaiza et al., 2006).

In Mrp2^−/−^ mice, the biliary excretion rate of BDG was about half than in wild types (Chu et al., 2006). However, in these knockout mice, no other efflux transporter, e.g., the sinusoidal efflux pump Mrp3, was activated by gene induction. Therefore, this mouse transgenic line corresponds to neither the Dubin-Johnson syndrome nor the Eisai hyperbilirubinemic rat. However, transfection of human MRP2 in Mrp2^−/−^ mice (Scheer et al., 2012) led to normalization of conjugated BR levels in both plasma and urine.

A study (Hayashi et al., 2012) showed that the drug 4-phenylbutyrate (4PBA) increased the cell surface expression and transport function of MRP2 in the MRP2-MDCKII cell line and of Mrp2 in the rat liver, without significantly changing the MRP2/Mrp2 mRNA level. This drug is used to manage enzyme defects of urea production, as in the syndrome known as Ornithine Transcarbamylase Deficiency (OTCD), in order to provide an alternative pathway for the excretion of excess nitrogen. Indeed, the same study observed that in OTCD patients, 4PBA increased hepatic MRP2 expression and decreased total bilirubinemia.

Another primary active transporter expressed at the canalicular side of the hepatocyte plasma membrane is the Breast cancer resistance protein (Bcrp). In Bcrp-knockout mice, no changes in serum BR were detected, showing that this protein has no relevant role in BDG excretion (Mennone et al., 2010). In these models, bile-duct ligation, which induces adaptive up-regulation of apical Mdr1 (P-gp) or sinusoidal Mrp4, did not change Bcrp expression nor serum BR. Partly different findings have been reported in an independent study (Vlaming et al., 2009), which demonstrated that Bcrp-knockout mice showed significant hyperbilirubinemia, mostly due to UCB.

#### Reflux of conjugated bilirubin into the blood in molecular defects of biliary excretion

In case of biliary obstruction or Dubin-Johnson syndrome, the serum levels of conjugated BR increase. The identification of Mrp3 as the main sinusoidal membrane transporter responsible for the reflux of conjugated BR into the blood has been demonstrated by using either knockout mice models or by finding upregulated Mrp3 expression as a side-effect of a diuretic drug, as a toxic effect of chemicals used in industry, or in critically ill patients.

Loss of either Mrp3 or Mrp4 in (^−/−^) null mutant mouse colonies caused no change of serum BR as compared to wild types. However, common bile duct ligation for 3 (Belinsky et al., 2005; Zelcer et al., 2006) or 7 days (Mennone et al., 2006) shed light on the different roles of these efflux pumps in experimental cholestasis. In Mrp3^−/−^ mice, a substantial decrease of serum conjugated BR levels was shown, as compared with controls, but levels of serum bile acids remained unchanged (Belinsky et al., 2005; Zelcer et al., 2006). By contrast, in Mrp4^−/−^ mice, serum levels of total BR did not change, whereas a 5-fold decrease of serum bile acids was observed as compared with wild type mice (Mennone et al., 2006). These findings suggested that that Mrp4 is a sinusoidal efflux transporter for bile acids, but not for bile pigments.

The same conclusion about the function of Mrp3 in promoting efflux of conjugated BR has been reached in studies aiming at clarifying the mechanism of toxicity of the diuretic drug tienilic acid (Nishiya et al., 2006). If administered to Eisai hyperbilirubinemic rats, a dose-dependent increase of total bilirubinemia coupled with up-regulation of (mRNA) Mrp3 and heme oxygenase-1 was recorded.

Treating mice with perfluorinated carboxylic fatty acids (PCFA) induced the hepatic expression of both Mrp3 and Mrp4, which increased levels of conjugated bilirubin and total bile acids in serum. Induction was mediated by activation of both peroxisome proliferator–activated receptor alpha (PPARα) and NF-E2-related factor-2 (Nrf2) (Maher et al., 2008).

Up-regulation of MRP3 and MRP4 on the sinusoidal membrane of hepatocytes was observed in post-mortem biopsies, taken from long-term critically ill patients (Vanwijngaerden et al., 2011). Liver biopsies revealed histological aspects of cholestasis. Pre-mortem blood samples showed predominantly conjugated hyperbilirubinemia and hypercholanemia. These findings consistently underline the role of these transporters in mediating active efflux of cholephilic anions into the blood.

#### The molecular barrier for bilirubin transport into the brain

At the level of the blood-brain barrier, P-glycoprotein has been shown to play a role in protecting the brain from accumulation of BR, as demonstrated by *in vitro* experiments reported above. The first study tested the role of P-glycoprotein in limiting the brain accumulation of BR. Thus, wild type and Mdr1a(^−/−^) null mutant mice received an intra-venous bolus of BR and their brain BR contents were measured 10 and 60 min after the infusion. Null mutants nearly doubled their brain BR, as compared to wild types (Watchko et al., 1998). This study contributed to demonstrating that P-glycoprotein could transport BR as an endogenous substrate. This observation was confirmed in experiments with wild type Sprague-Dawley rats (Hankø et al., 2003). Their brain-to-plasma BR ratio measured after an i.v. bolus of BR was higher if it was preceded by a short (10 min) infusion with P-glycoprotein blockers, like propanolol, verapamil, ceftriaxone, and rifampin.

## Discussion

### Outcome of the search strategy

The most unexpected outcome of having sourced PubMed by a multiphase search approach was the missing retrieval of at least 6 articles, tagged with correct MeSH terms.

These limitations can be overtaken by manual search, but wandering in the literature is subjective and inefficient. Another option might be the use of a different query, with “transport” instead of “transporter.” However, queries should straightly reflect the goal(s) of the systematic review. This review was meant at creating a list of BR membrane transporters, with no interest in articles dealing with other biological transport phenomena, such as albumin transport of BR in the blood or non-catalyzed diffusion of BR through biological membranes. Another implication of using generic query terms is that the burden of screening and eligibility checks may obfuscate the operational value of the automatic search.

We have also noticed articles that did not list “bilirubin” as keyword or MeSH term, in spite of reporting results about its analysis, which caused them to be out of the list of included papers. Authors should therefore be aware of a proper choice of descriptors of their works.

### Features of bilirubin membrane transport studies

Table 5 presents a synopsis of the outputs of our systematic review. Experimental models with increasing levels of structural and functional complexity (row 1) have been exploited to characterize the transporters (column 1) for BR and its conjugates. Discovery started historically with the most reductive experimental approach, i.e., BR binding assays on an isolated protein or binding/transport in plasma membrane vesicles. In the last decade, the development of knockout/knockin rodent models has enabled to investigate the functional role of membrane transporters *in vivo*.

**Table 5:** Synopsis of the experimental models used to study membrane transporters for bilirubin and its conjugates.

**Transporter**	**Protein**	**Vesicles**	**Cells**	**Tissues**	**Rodents *in vivo***	**Primates *in vivo***
Unknown		Adachi et al., 1990				
Organic Anion Binding Protein	Wolkoff and Chung, 1980					
BSP/bilirubin binding protein		Stremmel et al., 1983	Stremmel and Diede, 1990			
Bilitranslocase		Passamonti and Sottocasa, 1990; Passamonti et al., 1997	Passamonti et al., 2005b; Maestro et al., 2010	Terdoslavich et al., 2012		
OATP1A2, OATP1B1 /OATP1B, Oatp1b2			König et al., 2000; Cui et al., 2001; Briz et al., 2003; Wang et al., 2003; Chu et al., 2015		Chen et al., 2008; Lu et al., 2008; Zaher et al., 2008; van de Steeg et al., 2010, 2012, 2013; Watanabe et al., 2015; de Waart et al., 2016	Vanwijngaerden et al., 2011; Chu et al., 2015
MRP1, Mrp1		Jedlitschky et al., 1997; Rigato et al., 2004; Matsushima et al., 2017	Pascolo et al., 2001; Calligaris et al., 2006			
Mdr1/P-gp					Watchko et al., 1998; Hankø et al., 2003; Mennone et al., 2010	
MRP2, Mrp2		Jedlitschky et al., 1997; Kamisako et al., 1999; Rigato et al., 2004; Matsushima et al., 2017	Nakanishi et al., 2012; Matsushima et al., 2017		Hirouchi et al., 2005; Itani et al., 2005; Chu et al., 2006; Narvaiza et al., 2006; Scheer et al., 2012; van de Steeg et al., 2012; Watanabe et al., 2015	Hayashi et al., 2012
Oatp1a1 /Oatp1a4					van de Steeg et al., 2010; Gong et al., 2011; de Waart et al., 2016	
Bcrp					Mennone et al., 2010	
MRP3, Mrp3		Lee et al., 2004			Belinsky et al., 2005; Nishiya et al., 2006; Zelcer et al., 2006; Maher et al., 2008; van de Steeg et al., 2012	Vanwijngaerden et al., 2011
Mrp4					Mennone et al., 2006; Maher et al., 2008	Vanwijngaerden et al., 2011
MRP5		Matsushima et al., 2017	Matsushima et al., 2017			

*The transporters are listed according the publication date of articles first reporting relevant studies*.

Regarding the top 2 questions asked, i.e., “what transporters …?” and “what experimental models …?”, the extent or depth of knowledge about the listed membrane transporters can be all in all scored by the number of experimental models exploited to characterize a given transporter. Thus, rather than scoring single articles, we propose here to score the reproducibility of results on a given BR membrane transporter in multiple experimental approaches.

The champion in this review is the canalicular primary active transporter MRP2, investigated at 4 levels of biological complexity (i.e., vesicles, cells, *in vivo* rodent models and in humans) in 13 distinct studies. Next is the OATP cluster of sinusoidal transporters, investigated at 3 levels in 9 studies. Third is the sinusoidal transporter bilitranslocase, investigated at 3 levels in 5 studies. The “spillover” effect of the high intensity of MRP2 studies on knowledge related to the role of basolateral efflux transporters MRP3 and MRP4 benefited from the availability of genetically modified rodent models.

### Membrane transporters for bilirubin and its conjugates

#### What do we know now about bilirubin membrane transporters?

Table 6 lists the transporters studied in the 44 articles included in this review. With the exception of OABP and BBBP, all are annotated in the Transporter Classification Database (TCDB) (Saier et al., 2016). Of these, all but bilitranslocase have a gene name assigned by HUGO Gene Nomenclature Committee (HGNC) (Gray et al., 2015) and/or the Rat Genome Database (Shimoyama et al., 2015) and the Mouse Genome Database (Blake et al., 2017).

**Table 6:** Membrane transporters of bilirubin and its conjugates.

**Acronym**	**Organism**	**Names and synonyms**	**TCDB**	**RGD/MGI**	**HGNC**	**Bilirubin molecule**
OABP	Rat	Organic Anion Binding protein				Bilirubin
BBBP	Rat	BSP/bilirubin Binding protein				Bilirubin
BTL	Rat, human	Bilitranslocase. Bilirubin transporter	2.A.65.1.1			Bilirubin
Oatp1a1	Mouse, rat	Organic anion-transporting polypeptide 1a1. Solute carrier organic anion transporter family, member 1a1; Oatp1, Oatp1a1, Slc21a1.	2.A.60.1.1	Slco1a1	SLCO1A2	Conjugated bilirubin; bilirubin
Oatp1a4	Mouse, rat	Organic anion-transporting polypeptide 1a4. Solute carrier organic anion transporter family, member 1a4; Oatp1a4, Oatp2, Slc21a5; AI785519	2.A.60.1.6	Slco1a4	SLCO1A2	Conjugated bilirubin; bilirubin
Oatp1a5	Mouse, rat	Organic anion-transporting polypeptide 1a5. Solute carrier organic anion transporter family, member 1a5; Oatp3, Slc21a7		Slco1a5	SLCO1A2	Conjugated bilirubin; bilirubin
Oatp1a6	Mouse	Organic anion-transporting polypeptide 1a6. Solute carrier organic anion transporter family, member 1a6; 4930422F19Rik, Oatp5, organic anion-transporting polypeptide, Slc21a13	2.A.60.1.25	Slco1a6	SLCO1A2	Conjugated bilirubin; bilirubin
Oatp1b2	Mouse, rat	Organic anion-transporting polypeptide 1b2. Solute carrier organic anion transporter family, member 1b2; OATP2; Oatp4; lst-1; OATP-C; mlst-1; Oatp1b2; Slc21a6; Slc21a10; 7330442B20Rik		Slco1b2	SLCO1B1, SLCO1B3, SLCO1B7	Conjugated bilirubin
OATP1B1	Human	Organic anion-transporting polypeptide 1B1. Solute carrier organic anion transporter family member 1B1; LST1; HBLRR; LST-1; OATP2; OATPC; OATP-C; OATP1B1; SLC21A6	2.A.60.1.5	Slco1b2	SLCO1B1	Conjugated bilirubin; bilirubin
OATP1B3	Human	Organic anion-transporting polypeptide 1B3. Solute carrier organic anion transporter family member 1B3; LST3; HBLRR; LST-2; OATP8; OATP-8; OATP1B3; SLC21A8; LST-3TM13	2.A.60.1.12	Slco1b2	SLCO1B3	Conjugated bilirubin; bilirubin
OATP1A2	Human	Organic anion-transporting polypeptide 1A2. Solute carrier organic anion transporter family member 1A2; OATP; OATP-A; OATP1A2; SLC21A3	2.A.60.1.14		SLCO1A2	Conjugated bilirubin; bilirubin
Mdr1a	Rat, mouse	Mdr1a, mdr-3, MDR3, Evi32, multiple drug resistant 1a, P-glycoprotein, Pgp, P-gp, Pgy3, Pgy-3		Abcb1a	ABCB1	Bilirubin
MDR1	Human	CLCS; MDR1; P-GP; PGY1; ABC20; CD243; GP170	3.A.1.201.1		ABCB1	Bilirubin
Mrp1, MRP1	Rat, mouse, human	ATP-binding cassette sub-family C (CFTR/MRP) member 1a; MRP, Mrp1, multidrug resistance protein 1; Leukotriene C(4) (LTC4) transporter	3.A.1.208.8	Abcc1	ABCC1	Bilirubin
Mrp2, MRP2	Rat, mouse, human	ATP-binding cassette, sub-family C (CFTR/MRP), member 2; Cmoat, Mrp2, multidrug resistance protein 2	3.A.1.208.2	Abcc2	ABCC2	Conjugated bilirubin
Mrp3, MRP3	Rat, mouse, human	ATP-binding cassette, sub-family C (CFTR/MRP), member 3, MOAT-D, Cmoat2, multidrug resistance protein 3	3.A.1.208.9	Abcc3	ABCC3	Conjugated bilirubin
Mrp4, MRP4	Rat, mouse, human	ATP-binding cassette sub-family C member 4; (MRP/cMOAT-related ABC transporter; Multi-specific organic anion transporter B (MOAT-B)	3.A.1.208.7	Abcc4	ABCC4	None
Mrp5, MRP5	Rat, mouse, human	ATP-binding cassette sub-family C member 5; Multi-specific organic anion transporter C (MOAT-C); MRP5; SMRP; ABC33; MOAT-C.	3.A.1.208.15	Abcc5	ABCC5	Bilirubin
Bcrp1, BCRP	Mouse	Breast cancer resistance protein 1 homolog; Urate exporter	3.A.1.204.2	Abcg2	ABCG2	None

*Transporters are identified by codes of the TCDB, Transporter Classification Database; RGD, The Rat Genome Database; MGI, The Mouse Genome Database; HGNC, The HUGO Gene Nomenclature Committee*.

According to the TCDB, BR transporters are either Electrochemical potential-driven (coded as 2.A.) or Primary active (coded as 3.A.). According to HGNC, they are either solute carriers (coded as SLC) or ATP-binding cassette transporters (coded as ABC).

#### Primary active transporters mediate ATP-dependent cellular efflux of both conjugated and unconjugated bilirubin

These transporters have an intracellular ATP hydrolase domain and a transport site for the unidirectional substrate translocation. Their transport substrates are chemically heterogeneous, including many drugs. It is established that MRP2 determines transport of conjugated BR from the liver into the bile at the canalicular membrane. In case of cholestasis, up-regulation of sinusoidal MRP3 determines efflux of the conjugated pigment from the liver into the blood. At the level of the placental trophoblast and the blood-brain barrier, MDR1 or MRP1 mediate efflux of UCB toward the maternal or general circulation, respectively.

#### Electrochemical potential-driven transporters mediate bi-directional transport of bilirubin and its conjugates

These transporters transfer solutes up to their electrochemical equilibrium between membrane-separated compartments. In the liver, they are expressed at the basolateral membrane domain. They have wide substrate specificity and transport drugs as well.

#### Transporters of conjugated bilirubin

OATP1B1 and OATP1B3 transport BMG and BDG from the blood into the liver. *In vivo* studies with transporter-deficient mice lines have demonstrated a major role of OATP1B1/1B3 (van de Steeg et al., 2010, 2012) in promoting re-uptake of conjugated bilirubin from the blood.

#### Transporters of bilirubin

Less clear is the capacity of OATPs to mediate uptake of UCB from the blood into the liver. *In vitro* uptake studies have produced contradicting results, with evidence both in favor (Cui et al., 2001; Briz et al., 2003) and against (Pascolo et al., 2001; Wang et al., 2003) the hypothesis that UCB is a substrate of certain OATPs.

Population studies showed that single-nucleotide polymorphisms of either the gene *SLCO1B1* or the gene *SLC1B3* are associated with mild elevations of conjugated BR (Zhang et al., 2007; Sanna et al., 2009) or UCB (Sanna et al., 2009) in serum, respectively.

A deeper study with transgenic mice expressing OATP1B1/1B3/1A2 (van de Steeg et al., 2013) provided evidence in support of the concept that OATPs play a limited role in uptake of UCB into the liver.

Also, studies on the ontogeny of hepatic drug transporters (Mooij et al., 2014) indicated that the mRNA expression of OATP1B1 and OATP1B3 is 500–600-fold lower in human neonates than in adults. Considering that in neonates there is a rise of UCB in serum, it may be suggested that alternative membrane transporters for BR should be responsible for bilirubin uptake into the liver.

Furthermore, analyses of hepatic toxicity in humans and animals did not indicate strong association between OATP inhibition and hyperbilirubinemia, neither for humans nor for animals (Kotsampasakou et al., 2017b).

This does not mean that OATPs have little to do with membrane transport of bile pigments. First, their apparent limited role in determining UCB levels in serum might just indicate that BR and BDG are mutually competing for transport by OATPs, with prevailing affinity and occupancy of BDG in the transporter active site. Further clues might be found in other domains of the natural sciences. Two studies have shown that expression of OATP1B3 in the hen's oviduct and the shell gland confers the phenotype of depositing blue-shelled eggs, as a result of inclusion of biliverdin in the shell (Wang et al., 2013; Wragg et al., 2013). It may be therefore speculated that OATP1B3 may take part in the rapid and trans-compartmental (blood-liver) redox equilibrium of BR/biliverdin. The occurrence of the BR/biliverdin pair in human serum could be detected by advanced analytics (Martelanc et al., 2016).

Hence, UCB uptake into the liver must certainly result from redundancy and complementation of multiple membrane transporters, as it is for enzymes (Pandya et al., 2014). The synopsis in Table 5 shows indeed pieces of redundancy.

The membrane transporter bilitranslocase (BTL) has been characterized as a BR transporter in plasma membrane vesicles, where it has been kinetically characterized by the electrogenic BSP transport assay (Passamonti et al., 2010), with UCB as a competitive inhibitor (K_i_ = 0.11 μM) (Passamonti et al., 2005a). Time-dependent inhibition by either protein reagents or affinity-purified, anti-sequence antibodies showed that bilitranslocase has at least two apparent, high-affinity BR binding sites (K_i_ = 0.3 and 2 nM, respectively), located on extracellular domains of the protein and both targeted by 2 different anti-sequence antibodies (Battiston et al., 1998; Passamonti et al., 2005a). One of these antibodies inhibited cellular uptake of BR, added at 50 nM in an albumin-free solution (Passamonti et al., 2005b). It should be stressed that the electrogenic BSP transport assay measures the activity of a BSP transporter completely different from any other SLC transporter, as assessed by an extensive functional characterization of its competitive and non-competitive inhibitors (Zuperl et al., 2011) and as confirmed by BSP uptake tests in rat and human liver slices (Terdoslavich et al., 2012).

The troubled question about this transporter is its gene, which has not yet been characterized, as well as its mRNA, which corresponds to a large segment of the anti-sense strand of the gene that encodes for caeruloplasmin (Battiston et al., 1998; Passamonti et al., 2009) and follows therefore the unpredictable fate of sense-antisense RNA pairs. Yet, by applying a package of highly sophisticated computational approaches (Venko et al., 2017), the bilitranslocase primary structure was turned into a 3D membrane protein model that comprises 4 trans-membrane domains (Roy Choudhury et al., 2013), 2 of which interact and both are able to make a synergistic kink in the middle of the membrane, providing a theoretical structural requirement for substrate translocation (Roy Choudhury et al., 2015).

Both OATP1B1 (Wang et al., 2005; Mandery et al., 2012) and bilitranslocase (Passamonti et al., 2002; Karawajczyk et al., 2007) have the demonstrated capacity to transport dietary flavonoids that, by transport competition, may intermittently interfere with the fine blood-liver equilibrium of BR. It is obvious therefore that multiple, membrane transporters for UCB must coexist and concur to rapid and rate-unlimited BR uptake into the liver. It may be that the other BR-binding proteins in the list of Table 3, i.e., OABP (Wolkoff, 2014) and BBBP (Brandoni et al., 2012), or additional ones yet to discover, may contribute to BR uptake into the liver.

## Conclusions

This is the first systematic review of experimental studies on membrane transporters for BR and its conjugates. We selected 44 articles reporting *in vitro* and *in vivo* studies published from 1980 to July 2017. They reported about 11 electrochemical potential-driven and 8 primary active membrane transporters. Paradoxically, the remarkable advancements in this field have only confirmed the elusive mechanism(s) enabling UCB to diffuse into the liver as if no cellular boundary existed.

## Author contributions

SP designed the method, while JČ implemented and refined it. JČ created and managed the database of the PubMed search output, and the study features. SP and JČ independently screened and assessed the included articles. SP drafted the manuscript and JČ refined both the text and the database. JČ prepared tables and the figure.

### Conflict of interest statement

The authors declare that the research was conducted in the absence of any commercial or financial relationships that could be construed as a potential conflict of interest.
